# “It changed everything”: The safe Home care qualitative study of the COVID-19 pandemic’s impact on home care aides, clients, and managers

**DOI:** 10.1186/s12913-021-07076-x

**Published:** 2021-10-05

**Authors:** Pia Markkanen, Natalie Brouillette, Margaret Quinn, Catherine Galligan, Susan Sama, John Lindberg, Nicole Karlsson

**Affiliations:** 1 Department of Public Health, Zuckerberg College of Health Sciences, University of Massachusetts Lowell (UMASS Lowell), Lowell, MA USA; 2 Department of Health and Human Services, New Hampshire (NH) Environmental Public Health Tracking Program, Division of Public Health Services, NH, Concord, NH USA

**Keywords:** Home care, Home health care, Home care aide, COVID-19 pandemic, Qualitative methods, Infection prevention and control, Psychosocial demands, Personal protective equipment, Home care aide job retention

## Abstract

**Background:**

Home care (HC) services are crucial to the health and social wellbeing of older adults, people with disabilities, and the chronically ill. Although the HC sector is growing rapidly in the USA, there is high job turnover among the HC aide workforce. HC provides an important alternative to facility-based care, yet it has often been overlooked within the larger health care system: most recently, in COVID-19 pandemic planning. The objective of the study was to characterize qualitatively the impact of the COVID-19 pandemic on three key HC stakeholders: clients, aides, and agency managers.

**Methods:**

The study included 37 phone interviews conducted during April – November 2020: HC clients (*n* = 9), aides (*n* = 16), and agency managers (*n* = 12). All interviews were audio recorded and transcribed verbatim. Qualitative analysis of the transcripts followed the grounded theory approach. The interview transcriptions were coded line-by-line into hierarchical themes with NVivo 12 software which allowed weighting of themes based on the number of interviews where they were coded.

**Results:**

Fear of infection and transmission among HC clients and aides were strong themes. Infection prevention and control became the top priority guiding day-to-day business operations at agencies; sourcing adequate personal protective equipment for staff was the most urgent task. HC aides expressed concerns for their clients who showed signs of depression, due to increased isolation during the pandemic. The disappearance of comforting touch – resulting from physical distancing practices – altered the expression of compassion in the HC aide-client care relationship.

**Conclusions:**

The findings suggest that the pandemic has further increased psychosocial job demands of HC aides. Increased isolation of clients may be contributing to a wider public health problem of elder loneliness and depression. To support the HC stakeholders during the on-going COVID-19 pandemic, for future pandemic planning or other health emergencies, it is important to improve HC aide job retention. This action could also ease the serious care services shortage among the growing population of older adults.

**Supplementary Information:**

The online version contains supplementary material available at 10.1186/s12913-021-07076-x.

## Background

Home-based services are critical to the health and social wellbeing of older adults, people with disabilities, and the chronically ill [1–3]. In the USA, these services may be referred to as home health care (HHC) or home care (HC), depending on the degree of medical support provided. However, within these sectors, there is considerable overlap in the tasks performed by aides, the significant part of the home-based services workforce. In this paper, the term ‘home care’ (HC) refers to the full range of home-based services performed by HC aides to assist clients with activities of daily living, including personal care services (e.g., showering, toileting, mobilization) and homemaking services (e.g., cleaning, grocery shopping, laundry) [4].

HC is a rapidly growing industry in the USA and offers an important alternative to facility-based care; however, it is often overlooked as part of the larger health care system [5]. When older adults leave hospitals with a post-discharge plan of care at home, hospital readmissions can be reduced by more than 20% compared to patients without discharge plans [6, 7]. A recent study followed COVID-19 survivors (*n* = 1409) after their inpatient hospital care and upon admission to HHC services; it found that about a month later, after receiving home-based care, 94% of COVID-19 patients had been discharged and most showed improvements in symptoms and functional status [8]. The HC industry applied important lessons from earlier influenza outbreaks and a wide range of recommendations by key stakeholders were brought forward to inform pandemic preparedness planning in HHC and HC [9, 10]. Yet, these sectors were still overlooked nationwide at the beginning of the COVID-19 pandemic [5, 11–14] while challenged to function above capacity to provide home service visits [15].

If infected with the virus causing COVID-19, the home-based care population, especially those with respiratory illnesses [16, 17] or other comorbidities [18], is at increased risk of developing a severe health outcome. The Safe Home Care Project at University of Massachusetts Lowell, USA, is a research team that studies and promotes the safety, health, and wellbeing of the HC workforce [19]. In summer 2020, the Project team conducted a survey of managers in Massachusetts HC agencies (*n* = 94) to assess the impacts of COVID-19 on agencies, clients, and aides early in the pandemic. Most agencies (60%) provided services to clients with COVID-19 and three quarters of agencies reported that they employed HC aides who had tested positive for the virus, had been symptomatic, and/or quarantined [14, 20].

In the USA, care recipients can be called patients, clients, or consumers depending on the medical or social service system overseeing their care; in this paper, “client” refers to all care recipients [21]. There are many job titles for HC aides, based on their skillset and duties, for example, home health aide, personal care aide, personal care attendant, companion, and homemaker. In this paper, the term “HC aide” or “aide” refers to all aide jobs [2, 21]. HC aides are primarily women and increasingly people of color and immigrants [22]. HC aides are in high demand: the U.S. Bureau of Labor Statistics projects the employment of home health aides and personal care aides to grow 34% percent during 2019–2029 – a much faster growth than the average for other occupations [4].

Since the start of the pandemic, employers have expressed concerns that HC aides will leave their jobs because of fear of infection and that there will not be enough caregivers to replace those who leave [23]. Studies have reported on HC aides’ experiences during the pandemic [5, 13, 23, 24] pinpointing workers’ concerns about contracting COVID-19 as well as challenges in accessing appropriate personal protective equipment (PPE) and timely information about COVID-19 exposure and safety guidance. Studies have also highlighted the essential contribution of HC aides in working conditions that make them vulnerable to COVID-19 exposure and infection [22, 25, 26]. First, they cannot physically distance when performing hands-on client care tasks. Second, they typically service many clients a day, who may live in different types of settings such as private homes in houses and apartments, assisted living residences, nursing homes, and other settings [5]. In addition to impacts on health, the pandemic has brought financial concerns and other tradeoffs to HC aides [5, 24, 27, 28]. While ill, recovering, or quarantined, they are unable to work. Sick time is not provided for everyone and some may feel an intense financial pressure not to take time off [5, 14, 20, 29]. Many urban area caregivers rely on public transportation systems; due to operating less frequently during the pandemic, buses, trains or subways have become more crowded and not all commuters wear masks or physically distance [27].

### Objective

The objective of the study was to characterize qualitatively the impact of the COVID-19 pandemic on three key HC stakeholders: clients, aides, and agency managers. HC services are fundamental to the health and social wellbeing of older adults, people with disabilities, and the chronically ill. By addressing this fundamental need and building on existing literature, it is prudent to document and analyze the current pandemic experience from the perspectives of these three key stakeholders to inform policy and practice needs within the wider health care and social assistance system. This paper reports findings on (i) changes the pandemic imposed upon the daily lives of HC clients, (ii) top work-related COVID-19 concerns experienced by HC aides, and (iii) efforts that directors and managers at HC agencies and organizations prioritized to ensure the continuity of client services.

## Methods

### Remote research technique: phone interviews

This COVID-19 study was part of a formative qualitative phase of the larger intervention study of the safe Home care Project to improve the safety of HC workers and clients [30]. All study protocols and materials were approved by the University of Massachusetts Lowell Institutional Review Board (IRB), approval number 19–112-QUI-XPD. All procedures were followed in accordance with the ethical standards of the responsible committee on human experimentation (institutional and national) and with the Helsinki declaration of 1975, as revised in 2000. In addition to the IRB, the Massachusetts Elder Rights Review Committee approved the study methods and materials pertaining to HC client phone interviews. Due to the pandemic, in March 2020, the research team pivoted from in-person methods (i.e., focus groups, in-depth interviews) to remote phone in-depth interviews. The initial focus group and interview guides were modified for telephone administration*.* All updated guides included new questions on COVID-19 impacts. Appendix 1 includes selected COVID-19 questions asked in phone interviews

### Study population recruitment

The study reported here comprised 37 phone interviews (Table 1). The sampling strategy among all participant groups was purposive. It was deemed that 37 interviews were needed to reach the data saturation for the formative qualitative phase of the larger intervention study. Every participant completed an informed consent procedure either by signing an online consent form (all directors/managers) or agreeing to the verbal informed consent conducted on the phone preceding the interview (typically clients and aides).

**Table 1:** Safe Home Care COVID-19 qualitative study phone interview sessions conducted during April – November 2020 among participating Massachusetts-based HC agencies/organizations

Interview sessions #	Participant	Agency/Organization
**Directors/ managers at HC agencies and organizations (*n*** **= 12)**		
1.	Executive director	Private HC provider agency A
2.	Executive director	Private HC provider agency B
3.	Executive director	Elder service network organization
4.	Executive director	Provider agency network organization
5.	Clinical services director	Elder service agency A
6.	Clinical services manager	Private HHC agency C
7.	Care manager	Private HHC agency C
8.	Executive director	Private HHC agency C
9.	Executive director	Private HHC agency D
10.	Care manager	Private HHC agency D
11.	Care manager	Elder service agency B
12.	Care manager	Elder service agency B
**Home care aides (*n =*** **16)**		
HCAs 1–8	Home care aides	Employed at private HC provider agency A
HCAs 9–16	Home care aides	Employed at private HC provider agency B
**Home care clients (*n =*** **9)**		
Clients 1–9	Clients	Receive HC services through elder service agency B

Twelve (12) directors and managers from seven different HC agencies and organizations were invited to participate through a purposive recruitment strategy and were interviewed during April–November 2020. Most of these participating provider agencies and organizations had a pre-existing research partnership with the Safe Home Care Project. One provider agency was newly recruited in 2020. Potential director and manager interviewees were contacted by email either directly or through an agency study liaison who forwarded a phone interview invitation and one-page factsheet on the interview process. HC agency directors, managers/supervisors, and clinical staff were eligible to participate.

Sixteen (16) HC aides recruited from two HC agencies were interviewed during September–October 2020. These two HC agencies were selected purposively. In one of these agencies, 16 volunteers had signed-up for a focus group that had to be cancelled due to the pandemic. The agency manager supported the remote phone interviewing method, and the Safe Home Care Project mailed (through U.S. Postal Service mail delivery) a recruitment packet to all these earlier 16 volunteers. The recruitment packet consisted of an informational brochure about the phone interview process, a cover letter, and volunteer reply form accompanied with a postage-paid return envelope addressed directly to the Safe Home Care Project. In total, nine volunteer reply forms were received and eight of them participated. In another agency, similar phone interview recruitment packets were mailed to 30 eligible participants of whom 8 returned the volunteer reply forms and participated.

Nine (9) HC clients recruited from one elder service agency were interviewed during September 2020. This participating service agency was recruited through a membership network organization. With assistance of the agency study liaison, an email recruitment of eligible clients was attempted first; however, no volunteers were obtained. In the second attempt, the study liaison mailed 50 recruitment packets, similar to that described above, to eligible clients. The selection criteria were as follows: currently active or recently suspended HC clients, English speakers, and capacity to provide a verbal informed consent. The Safe Home Care Project received volunteer reply forms from ten clients of which nine participated.

### Data collection and analysis

The senior qualitative investigator (PM) conducted all phone interviews, which lasted no more than 1 h. All sessions were audio recorded and transcribed verbatim. The participants received a $40-incentive check by mail post-interview. They also received a copy of their typed interview session transcript by postal mail (clients, aides) or by email (directors/managers) and were given an opportunity to provide feedback.

Qualitative analysis of the transcripts followed the grounded theory approach [31, 32]. The computer-assisted qualitative analysis process followed a technique similar to that utilized in earlier Safe Home Care Project qualitative and mixed-methods studies [1, 21, 33, 34]. The phone interview transcripts were coded line-by-line into hierarchical themes – up to 5 levels – with NVivo 12 software. This software allowed for weighting of themes based on the number of different interviews where they were coded. A team member trained in qualitative methods conducted the first coding round of all transcripts. The senior qualitative investigator verified these codes by cross-referencing each verbatim transcript quote and corresponding coded theme. If necessary, modifications were applied. The parent theme (COVID-19) and secondary child themes were determined a priori based on the interview guide questions related to the COVID-19 experience. The grandchild and associated subthemes were identified from the transcripts using specific coding techniques to capture the participants’ narratives.

### Voluntary demographics survey

All interviewees were invited to complete a voluntary, anonymous survey to report demographics information (gender, age, race, and ethnicity). Directors and managers received the Qualtrics online link via email. HC aides and clients received the 1-page demographics survey (Appendix 2) by postal mail together with a typed transcript of their phone interview session. A postage-paid return envelope was provided to return the survey and any other feedback about the phone interview process to the Safe Home Care Project.

## Results

### Participant characteristics

Twenty-four out of 37 participants responded to the demographics survey (Appendix 2) of the study: 10 aides, 8 directors/managers, and 6 clients. All reporting participants self-identified as female. The average age of all participants was 59, ranging from 30 to 76 years. Mean age was similar among aides (56; range: 30–76) and managers (53; range: 37–68), while clients were generally older (71; 63–76). Clients reported receiving HC services for an average of 5 years, ranging from 1 to 10 years.

For the 24 participants who returned the survey, aides were Black (*n* = 4) or White (*n* = 5), while all managers (*n* = 8) and clients (*n* = 6) reported their race as White. Among these participants (managers, aides and clients), most identified as Not Hispanic/Latino (*n* = 15, 63%) and one participant (an aide) identified as Hispanic/Latino (*n* = 1, 4%). There were significant missing values for this ethnicity question (*n =* 8, 33%).

Aides worked an average of 17 years in HC, ranging from 3 to 31 years. The most common job title was home health aide (*n* = 9, 60%), followed by homemaker (*n* = 3, 20%). Aides also reported multiple job titles, such as “home health aide” and “hospice aide” (*n* = 1), or “Certified Nursing Assistant” (*n =* 1). Half of directors/managers were executive directors (or equivalent titles) and half held either a clinical services director/manager or care manager position at their agencies or organizations (Table 1).

### The COVID-19 pandemic impact on home care clients

Table 2 summarizes the most frequently coded themes on the pandemic’s impact on the daily lives of clients (*n* = 9).

**Table 2:** Most frequently coded themes and their subthemes on how the pandemic affected the daily lives as reported by clients (*n =* 9) during phone interviews of Safe Home Care COVID-19 qualitative study

Themes	Subthemes	Frequency coded
**Fear of risk of infection**	• Going out only when necessary o Feeling worried and being more alert when going out o Washing clothes each time after going out o Food shopper does the groceries • Concerned about being a carrier and spreading the virus • Washing hands more often	***
**Less physical connection with others**	• Physical distancing guidelines • Communicating on the phone more than seeing each other in-person • Unable to interact with other residents like before pandemic • Family member can no longer assist with compression stocking removal	***
**Little change prior to pandemic**	• Did not go out much prior to pandemic • Calls and visits from family to check in/ help out • Neighbor visits and wears a mask • Client continues receiving treatments at hospital	***
**Telehealth use**	• Video telehealth technology not commonly used o Connection problems • The phone is the most used communication tool o Communication with HC-service case managers, nurses	***
**Wearing masks**	• Client wears a mask for medical appointments • Client cannot wear a mask due to the health condition • Difficulty hearing others speak • Difficulty reading lips when HC aides and other people wear masks	**

Coding frequencies (*n =* 9): *** 5 or more participants; ** 2–4 participants.

#### Fear of infection

Fear of infection was reported in all nine client interviews. A root cause for this fear may be due to variations in how other individuals respond to the seriousness of the disease or differing levels of cooperation and participation regarding guidelines and precautions. One client expressed her worries as follows:

Oh, I’m paranoid about it a little bit. I’m scared about it, but you know, I don’t go into it, but I wished the other people would start listening to other people. But I think if you just do what they warn. You know, wear the mask … be six feet apart and quit rushing, people … it’s dangerous out there*.*

- HC client interview #4

One client reportedly suspended HC services due to fear, only to reinstate them several months later. Another client had suspended services due to household-to-household virus transmission concerns; during the time of the interview, she was considering resuming them. Clients were also worried about being carriers and transmitting the virus to others. Many clients left home only for necessary events (e.g., medical appointments) and reported being afraid and worried when outside the home. One client described her feelings as follows:

I don’t think I’ll be out there, out and about… And I’d rather avoid being near people that might be spreading germs. So I kind of stay in my house. I don’t go out anywhere. I’m up to my daughter’s occasionally to go outside … that’s about my social scene since this past June*.*

- HC client interview #7

#### Physical distancing, mask wearing, and communicating by phone

HC clients described how the pandemic affected their services in three primary ways: (i) aides wear a mask all the time and maintain physical distance unless performing hands-on care tasks, (ii) case/care managers utilize phone communication rather than in-person visits to clients’ homes, and (iii) meal deliverers leave groceries outside the door rather than bringing them inside and putting them away. On one hand, clients appreciate caregivers wearing masks, gloves, and other personal protective equipment (PPE). Nevertheless, it was expressed that mask-wearing compromises the client-caregiver communication:

Well, before, you know, the COVID-19, everything and everybody was happier and more cheerful, and excited, more relaxed. And now, it’s just so scary and people are confused and it’s hard to read lips when people got masks on. I have problems with hearing, so I read their lips, and I can’t. So, it’s hard. I have to keep saying, what’d you say, what’d you say?

- HC client interview #8

Despite the fear, mask-wearing, and physical distancing measures, most clients (7 of 9) felt that their fundamental needs were met. By adding considerable effort and time, caregivers managed to perform the same duties as before the pandemic and arrived punctually to each service visit. Video “telehealth” was not commonly used by most interviewed clients (7 of 9). The phone was the most used communication tool with case/care managers and other care providers who could service clients remotely. Only two clients utilized video communication platforms to meet face-to-face with their providers and they both had experienced technical glitches (e.g., videos freezing or difficulties in hearing one another). The phone was considered the most useful and reliable all-purpose communication technology.

### Top COVID-19 concerns among home care aides

Table 3 lists the top COVID-19 concerns reported by HC aides.

**Table 3:** Most frequently coded themes and their subthemes on the top work-related COVID-19 concerns as reported by HC aides (*n* = 16) in phone interviews of Safe Home Care COVID-19 qualitative study

Themes	Subthemes	Frequency coded
**Aides concerned about becoming infected with COVID-19 from clients**	• Clients as possible carriers • Bringing the virus home to her own family • Not receiving an alert about a COVID-positive client • Being vigilant with everything that needs to be touched • Clients may not wear masks, especially during personal care tasks • Consequences of becoming sick and being unable to work o Financial and livelihood hardships	***
**Aides concerned about wider occupational and community COVID-19 transmission**	• Visitors exposing clients to COVID-19 o Many visitors entering clients’ homes, often at the same time ▪ Family members, friends, neighbors, caregivers ▪ Visitors who do not wear masks or distance • Being a carrier between clients’ homes and exposing elderly clients, others to COVID-19 • Despite heeding precautions, the stress of transmission remains	***
**Aides empathize with elderly clients who cannot go out of the home**	• Clients showing signs of depression from distancing and isolation o Councils on Aging, senior centers, other activity places closed o Clients getting bored while being stuck at home • Social distancing has pronounced effect on dementia clients • Clients are anxious and worry a lot	**
**Distancing affects compassion & socialization aspects of HC**	• Unable to comfort clients with physical connection • Distancing creates barriers between people • Some clients do not understand why physical connection is not allowed	**

Coding frequencies (*n =* 16): *** 8 participants; **3–5 participants.

#### Concerns about virus transmission

During the study period, none of the HC aides reported caring for known COVID-19 positive clients. All aides monitored their symptoms and remained alert for symptoms among their clients. Prior to the first care visit each day, half of interviewees reported filling out a COVID-19 symptom questionnaire online. All interviewees reported that their employing agencies supplied them with needed PPE and provided instructions about the necessary precautions. All HC aides must (i) wear a clean, previously unused surgical mask at each client visit, and (ii) physically distance at least six feet except during hands-on personal care tasks. In addition to the mask, half of interviewees also wore either a face shield or goggles. They already practiced proper hand hygiene and wore gloves prior to the pandemic. Now they described a constant state of caution and attentiveness regarding the possibility of virus transmission. Two HC aides discussed the situation as follows:

Like how you walk away, come back to the house, it’s very stressful that, oh, we’re careful to put our mask, wash our hands, put the gloves before you do the care.

- HC aide interview #15

You have to be very careful at work with things you touch, make sure you keep sanitizing. I was before, but now you have to be extra careful in elevators and door handles when you go in a building*.*

- HC aide interview #3

If infected, financial hardship was also mentioned as one of the top concerns:

I’m concerned, because I’m not rich, but hard if I catch anything… [I have] to be careful [with] everything I touch*.*

- HC aide interview #5

Interviewees provided varying reports as to how infection prevention and control (IPC) had changed since the pandemic began. Some thought wearing a mask was the biggest difference while others thought IPC practices were significantly changed*.* Some experienced discomfort from wearing a mask and face shield albeit understanding that the discomfort had to be endured, for example:

…putting on the PPEs and wearing a mask and the face shield, and you are going to showering the clients, you find the shield, they can get sometimes foggy, and sometimes in the summer, it has been so hot, so when you are sweating in the mask, you know, it’s just bit uncomfortable. But you have to endure it*.*

- HC aide interview #9

And no matter what I do with the mask, I pinch it on my nose, I wear glasses and they’re always fogging up. And then the water or the sweat is, and my hair’s just drenched and it’s just getting into my [eyes], or my glasses. I can’t see and it’s horrible…It’s something we have to do*.*

- HC aide interview #13

The amount of time spent cleaning and disinfecting during care visits did not change according to half of interviewees while approximately half (7 of 16) reported cleaning more frequently. Most HC aides (10 of 16) explained that clients or their family members purchased the cleaning products used during service visits or aides purchased products as part of their homemaking service visits which were reimbursed by clients.

#### Impact on empathy and expression of compassion

When aides were asked how COVID-19 was affecting the lives of their clients, 5 of 16 interviewees brought up concerns about isolated clients, in particular the ones who showed signs of depression. Contributing factors included a combination of elderly age, limited mobility, social distancing, loneliness, anxiety, stress, and closing of activities. Clients were no longer engaging in social activities they used to enjoy like meeting friends outdoors and having meals together. Physical distancing resulted in changes to interactions between clients and family members. Senior Centers and Massachusetts Councils on Aging^1^ sites had closed. Aides suggested coping strategies to clients including going for a walk if mobility allows, listening to music, or negotiating with the case manager for extra care time so aides could assist them to go outside. One of the interviewees described how she felt about this:

They’re very depressed because a lot of them used to go to Bingo. They used to go outside and especially during the summer to sit with their friends. So, it’s making them very depressed, very stressed …

- HC aide interview #8

Aides reported their mask use had saddened some clients: “*It’s changed a lot where we have to wear masks… Some of the clients become saddened. They like to see our smiles.”* Isolation was reported to be problematic for any client who lives alone. For clients with dementia, the pandemic could be a frightening experience:

Plus some of the clients, they don’t recognize you. You know, sometimes they have dementia … so it’s hard for them to recognize you with a mask… they can get afraid, or they think it’s something else*.*

- HC aide interview #11

HC aides also reported that the pandemic had curtailed physical touch and compassion from the job. This experience was described as *upsetting, disturbing,* or *disappointing.* HC aides explained that they may be the only people clients see now regularly in-person. Unless performing personal care tasks, aides can no longer comfort clients with physical connection. One aide discussed as follows:

…most of the time we’re the only face that they see after their family comes maybe on the weekend. So, sometimes they get lonely… before if somebody is feeling sad, I can go and give them a hug. I can’t do that anymore. When somebody was feeling down in the slopes, we would go for a walk and I could actually hold their hand and if they were, want assist, I can’t do that anymore. So, I think the touching part of the compassionate part has been cut at our end*.*

- HC aide interview #12

### Perspectives of HC agency directors and managers

Table 4 summarizes the most frequently coded themes from HC agency director/manager interviews on priorities that they had to address.

**Table 4:** Most frequently coded themes and their subthemes from director/manager interviews (*n* = 12) on the impact of COVID-19 on HC agencies during phone interviews of Safe Home Care COVID-19 qualitative study

Themes	Subthemes	Frequency coded
**PPE sourcing for staff a high priority**	• Initial PPE shortage, donations helped at the beginning • PPE availability sufficient now but requires continuous effort o PPE sourcing may be one person’s full-time job • Masks required for HC aides during care visits o Surgical disposable masks most commonly used o N95 respirators, envo® masks for direct COVID-19 care • Gloves required for HC aides during care visits • Face shield, goggles may be required during care visits o HC aides not allowed to wear cloth face coverings during care visits	***
**Managing COVID-19 positive/ symptomatic HC clients and staff**	• Tracking and monitoring HC staff, client health symptoms o Require HC staff, clients to self-monitor for COVID-19 symptoms and self-report • Change care model to isolate suspected and positive COVID-19 clients and reduce risk to HC aides while continuing to provide necessary care tasks o Create a reporting chain and alerting system for suspected and positive COVID-19 clients, HC staff • HHC agencies with skilled nursing care o Direct care of positive cases in the home o Anticipate post-acute care after hospital discharge • HC agencies contracted for hands-on personal care with aides o Higher visit rates paid to agencies for positive cases • Ensuring adequate PPE for staff caring for the positive cases • Situational awareness team at an elder service agency upon identifying a positive case o Communicates remotely, follows protocol for care decisions	***
**Training and communication with HC staff**	• Remote communication methods during the pandemic • In-service training for HC aides offered remotely online or through self-study assignments • Staff may be technologically challenged o Challenges in communicating information electronically to staff o No company email for staff – personal email only • Brief, scheduled visits allowed in the HC office o PPE, accumulated mail pick-ups o HC aides miss one-on-one interaction with colleagues	***
**HC aide staffing/ retention challenges**	• HC aide staffing, visit scheduling challenges o When caregivers quarantined o When clients first suspend and later resume services • Difficult to retain HC aides o Underfunded HC industry	**
**Clients reaction/ behavior**	• Initial fear o HC service visit cancelled, suspended, or referrals reduced o Meal services to clients increased • Mask wearing among clients varies	**

Coding frequencies (*n =* 12): *** 7 or more participants; **5–6 participants.

#### Changes in daily business operations to manage COVID-19

The pandemic pushed all agency administrators to work remotely from home, which added another layer of challenge to manage the emergency. During the first few weeks, case/care managers called every client by phone and then developed a list of clients to be followed up by phone on a weekly basis. One director described the experience at the beginning:

It was really difficult, and I really felt like you couldn’t keep a handle on everything. And so much information being passed along and being shared that you didn’t know if you’d taken care of it or not… it’s gotten better … we’re communicating better with one another, and we’ve also put things in place so that we’re not scrambling… I don’t think anybody saw anything as massive as this coming*.*

- HC manager interview #5

IPC became the highest priority to guide day-to-day operations. Managers focused on ensuring that staff at all levels were safe, and had enough and appropriate PPE to provide care to clients both with and without COVID-19 diagnoses. This required an organized effort from all stakeholders to influence, interpret and implement Massachusetts state government policy. At the beginning of the pandemic, there was a serious shortage of PPE. Agencies were concerned about how much PPE would be needed and how long these items would last. Two interviewees reported the following in May 2020:

The issue of having enough PPE is a huge issue for us and we’ve been working with the State and trying to get both the administration and the legislature and trying to make sure that homecare workers are listed as not only essential workers but get priority for supplies of PPE*.*

- HC manager interview #3

Maybe until mid-April [2020] there was really, the home care space was not being prioritized for PPE… the initial PPE document actually listed home care workers as like a small part of family caregivers, so you know, if they have PPE that’s great, but they [were] not considered really Tier 1 for PPE*.*

- HC manager interview #4

All HC aides had already been using gloves and heeding proper hand hygiene during the client visits. However, masks, goggles, and gowns were not commonly used prior to the pandemic and almost no one was using N95 respirators or face shields. During the first two months of the pandemic, agencies focused on understanding what PPE was needed and sourcing it. To add to the complexity, guidance on PPE changed continuously as the pandemic moved along. As of June 2020, an agency director described their situation:

… We do currently have enough access to PPE supply. I think that is a product of having virtually one person sourced for PPE as the primary sort of full-time job. You know, we’ve had multiple donations from multiple companies … of face shields and masks, and we have reached out to various vendors, which has been a full-time job, to try to source legitimate PPE.

- HC manager interview #8

#### Staff training needs

Even though IPC was already part of the annual training program at all provider agencies prior to the pandemic, staff training on COVID-19 precautions became among the highest priorities. This included developing educational materials, procedures, policies, and communicating all of it out to staff. Even though many agencies have websites where educational materials can be made available and emailing was common, information access challenges remained. A provider agency director explained in May 2020:

…our staff is on the road, so they don’t have a PC in front of them, or a laptop … a lot of our staff are technology challenged… information is there, but how they get the information. How do we communicate with our staff where a lot of them, we don’t have company email? [T]hey have personal emails, but not everyone… getting the latest information, even though there are ways to do it, to get our staff to know how to do it, and have access to it, is difficult*.*

- HC manager interview #2

#### Challenges in staffing and retaining caregivers

Screening the health symptoms from both clients and staff is time consuming. Even for clients whom managers knew “*like the back of your hand,*” it was important to ask screening questions because health conditions change quickly. When someone was identified or presumed positive and quarantined, HC staff scheduling became complicated, for example:

I would say the top issues would be scheduling…making sure our caregivers and our clients are not crossing kind of types of service… if you’re working internally in independent living, you can’t go see anybody else, anywhere else… [and] then on top of that, we’re very diligent and probably over cautious in terms of anybody who has symptoms… caregivers, if they have a symptom, they are out for two weeks*.*

- HC manager interview #9

Managers both at provider and elder service agencies described that many of their clients had suspended HC services at the beginning of the pandemic due to the fear of infection. Staff scheduling challenges compounded when clients suspended and resumed services. Resuming services may take time and the client can lose the regular HC aide provider:

[A] lot of people did suspend their services at the beginning of the pandemic, but they’re still open with us… But another issue is when they want to resume their services. It’s taking longer to resume them just because there aren’t a lot of aides available right now…

- HC manager interview #12

Directors at provider agencies described the general problem about the underfunded HC industry and how it has been affecting the retention of caregivers. During the pandemic, HC aides are performing essential tasks. Yet they can rarely secure fulltime hours at one agency or be able to earn a living wage. One director described the difficult situation as follows:

Especially now you see my workers are considered essential services out there and yet they’re just not paid enough to really show the value of the service that they’re providing… They really are out there… jeopardizing themselves to provide this essential service and they’re physically making barely above minimum wage to do that… then to try to balance that and still fund an agency at the same time leaves very little funding to keep an agency moving forward financially too. So it really is not funded anywhere near where it should be*.*

- HC manager interview #1

### Triangulation across different participant groups

Each participant shared COVID-19 pandemic experiences from distinct perspectives that may have reflected their stakeholder role, health condition, social situation, or professional responsibilities. The client’s experiences affect the care setting: the home. Although some of the client interview questions were asked without an explicit reference to HC (Appendix 1), these too elicited relevant data for the delivery of HC services (e.g., clients unable to read lips when aides wear masks).

The HC aide (i.e. the frontline care provider) balances job duties, agency policies, and the daily reality of a home-work environment controlled by the client or client’s family. The narratives of HC aides blended (i) changes to the nature of the client-aide care relationship due to the pandemic; (ii) how the aides’ safety and emotional wellbeing was affected by client’s health (e.g. dementia, depression, limited mobility), daily habits (e.g., staying at home all day), and social interactions (e.g., multiple visitors in the home); and (iii) how an agency’s IPC framework could both help (e.g., adequate PPE) and complicate (e.g., communication difficulties with client when wearing masks) the care delivery.

Agency directors and managers provided the care delivery perspective of the HC industry including pandemic-related clinical practice, business management, and rapidly changing public health policies. Their narratives emphasized IPC practices, staffing challenges, and underfunding of the HC industry.

All three groups were concerned about virus transmission although their apprehensions were narrated differently. Discussions reflected emotional stress due to the *massive change* the pandemic had on day-to-day business operations, HC service delivery to clients, and uncertainty related to the intensity and duration of the pandemic.

## Discussion

### Fear of infection and virus transmission

The results showed considerable fear of infection and virus transmission among both HC clients (Table 2) and aides (Table 3). Many clients cancelled or suspended their HC services. HC aides reported being in a constant state of caution and attentiveness regarding the possibility of virus transmission. Although directors/managers did not explicitly say “fear” or “being scared”, their responses reflected worry and concern about the unpredictable course of the pandemic. The fear of infection supported the findings of the aforementioned cross-sectional survey among Massachusetts HC agency managers (*n* = 94) that assessed the impact during the three first months of the pandemic (March – May 2020) [14, 20]. In the survey, the majority (81%) of participating agencies reported that HC aide visit hours had declined: nearly all (99%) indicated that clients were unwilling to have aides come into their homes due to infection concerns and about three quarters (74%) reported aides were unwilling to visit clients’ homes due to infection concerns [14, 20]. A qualitative analysis from a nationwide survey of HC aides (*n* = 1204) found that during early weeks of the pandemic, HC aides experienced either decreased service requests from clients or requests to take on additional clients due to staffing shortages [11]. Another qualitative study using HC aide journaling and interviews identified themes related to the fear of infection and general transmission of COVID-19 [13].

When examining the results broadly, surveys of HC aides in New York [24] and Michigan [13] as well as surveys of HC agencies nationwide [35, 36] have reported similar findings with our study. These include the lack of or difficulty sourcing PPE, concerns over transmitting or contracting COVID-19, grappling with decisions to work with increased exposure risk or face financial hardship, and accessibility issues regarding telehealth.

### Infection prevention and control enabling the continuity of services

The pandemic forced more extensive IPC programs at elder service and provider agencies to immediately address such priorities as sourcing PPE, workforce training and communication, and managing symptom screenings of both clients and staff which directly affected staff scheduling (Table 4). Until mid-April 2020, the HC sector was not prioritized for PPE by the Massachusetts government and limited supplies were diverted elsewhere. Other studies have also reported a lack of unified messaging from federal, state and local governments early in the pandemic which complicated implementing IPC protocols [11].

IPC programs often require updates as new public health guidance becomes available, including accounting for the importance of asymptomatic virus carriers, addressing the spread of the virus through aerosol transmission, taking precautions with virus variants that seem to spread more rapidly and may be more virulent, and finally, developing and implementing vaccine administration programs. Nonetheless, the IPC programs have enabled the continuity of HC services to clients during the frightening and stressful time: in this study, most participants reported that they had found ways to navigate the challenging circumstances, although mainly by contributing more time and effort. COVID-19 vaccine administrations had not yet begun during the interviews of this study. As of early March 2021, a national survey of U.S. frontline health care workers (*n* = 1327) found that slightly over half (52%) of frontline workers and about a quarter (26%) of HHC workers had received at least one dose of a COVID-19 vaccine [37].

Literature widely documents promising benefits of telehealth and how the pandemic has certainly accelerated its use [15, 38–40]. However, not everyone can realize its benefits equally in HC. First, hands-on personal care tasks cannot be performed remotely. Second, only a limited number of clients can easily access video conferencing tools. The client participants in this study reported that the phone remains the most reliable and efficient communication method. Currently, telehealth seems to benefit those who have financial resources and/or technical capacities to access video conferencing technologies. The limitations of telehealth are also not yet well-characterized [41].

### The pandemic and the changed nature of the aide-client care relationship

HC aides interviewed in this study empathized with and expressed concerns about clients showing signs of depression, largely due to the increased isolation (Table 3). Director/manager interviews also confirmed the increased isolation among clients. Published studies report extensively on the worsening isolation among elderly during the COVID-19 pandemic [39, 42, 43] and specifically how clients with dementia or other cognitive impairments may be affected [44–47]. Aides may be the only people with whom clients interact regularly. The disappearance of comforting touch – as dictated by the physical distancing practices – has altered the compassion aspect of the client-caregiver relationship. Clients with impaired hearing who normally rely on reading lips cannot do that now that caregivers wear masks. This too affects the communication and core care relationship between the client and the caregiver.

The pandemic impacts on the client-caregiver care relationship have increased HC aides’ emotional job demands. Pre-pandemic studies have shown that a significant factor for increased job satisfaction among HC aides has been the feeling that their work is genuinely appreciated by clients and that aides and clients can develop positive, meaningful relationships [1, 2, 21, 34, 48–50].

### Home care aide demand exceeds the supply

The demand for HC aide services has been exceeding the supply for over a decade and the current pandemic has further deteriorated the problem. The current shortage of HC aides was confirmed by agency directors and managers interviewed in this study. In the USA nationwide, HC aides’ low compensation for their work continues. According to the U.S. Bureau of Labor Statistics, the 2020 median annual salary of aides was slightly over $27,000 [4] which is virtually the same as the poverty line for the family of four [51]. In addition to existing low pay, lack of benefits, and inconsistent work hours [4, 22, 52], the current pandemic has further augmented caregivers’ psychosocial burden. This can accelerate the HC aide job turnover and deepen the existing shortage of caregivers.

### Recommendations to support HC stakeholders

To continue managing the current pandemic long-term and planning for future pandemics or other health emergencies, there are recommendations for action that can support HC stakeholders. First, it is critical to address the shortage of HC aides and reduce job turnover among them. This is the right time to increase HC aides’ wages to a level of living wage. The U.S.-based economic analyses about paying direct care workers a living wage [12, 53, 54] have shown that the accrued savings and gains (e.g. tax revenues, economic spillover, turnover reduction, productivity gains) outweigh the costs (e.g. wages, payroll tax, health care/insurance). A report from New York emphasized that significant increases in HC sector wages and benefits will require new public funding because blanket wage increases would problematically burden the caregiver employing agencies and households [53]. Nonetheless, new public funding has been recently made available short-term through the federal COVID-19 relief legislation: the $1.9 trillion American Rescue Plan Act of 2021 directs a 10% increase – approximately $12.67 billion -- for home and community-based services for April 1, 2021 to March 31, 2022 [55, 56]. Also, the proposed American Jobs Plan has called the U.S. Congress to invest $400 billion to *solidify the infrastructure of [the] care economy by creating jobs and raising wages for essential home care workers* [57].

Second, recognizing, and respecting direct care workers as members of the broader health care team would improve jobs and likely increase the job retention [5, 52, 58]. This has been described as overcoming the *cultural or attitudinal* challenge among the key actors in the wider long-term care system who have little respect for the direct care workforce [58].

Third, the increased isolation of HC clients during the pandemic may be contributing to elder loneliness and depression. This wider public health concern would need targeted interventions, for example, through (i) social assistance and public health programs and policies, and (ii) community-based participatory action research. Promising examples of such intervention programs to decrease social isolation have been implemented [59, 60].

### Limitations of the study

This study recruited HC aides from two provider agencies and clients were recruited from one elder service agency. Therefore, caution should be used in generalizing the findings. For example, colleagues from other states reported a serious lack of PPE availability for HC aides and were surprised to learn that Massachusetts HC agencies were able to procure N95 respirators, albeit with significant challenges. This study focused only on HC aides hired by agency employers and did not include aides hired directly by clients. The pandemic limited the research techniques to remote methods and the phone was the most reliable and accessible option for interviews. For this study, the demographics survey was distributed either via postal mail or online and yielded a lower response rate than in previous qualitative studies where the survey was handed to participants in-person.

## Conclusions

This study characterized qualitatively the impact of the COVID-19 pandemic on HC clients, aides, and agency directors/managers in the state of Massachusetts. Even though the fear of COVID-19 infection is still high, the new forms of work organization and IPC policies and practices have provided a path for continued HC services. However, the pandemic has had the unintended consequence of undermining the nature of the client-caregiver care relationship, rendering it less compassionate due to physical distancing and masking requirements. HC aides’ psychosocial burden has increased during the pandemic.

## Supplementary Information

**Additional file 1.**

**Additional file 2.**

## Data Availability

The data for this study is confidential as required by the two IRB approvals. To protect the anonymity of the participants, the data is not publicly available. Information about the data, methods, and the study in general can be requested from the corresponding author.

## Abbreviations

HC: Home care
HHC: Home health care
IPC: Infection prevention and control
IRB: Institutional Review Board
PPE: Personal protective equipment

## Footnotes

1 The Massachusetts Councils on Aging (MCOA) functions to provide senior citizens and their families access to resources, as well as social, educational, health, and recreation programs. Additionally, MCOA serves as an avenue to the state and local network of elder services. Further information available at: https://mcoaonline.com/
