# Supplementary material for: “It changed everything”: The safe Home care qualitative study of the COVID-19 pandemic’s impact on home care aides, clients, and managers
Source: BMC Health Serv Res. 2021 Oct 5;21:1055. doi: 10.1186/s12913-021-07076-x (PMC8491760; doi:10.1186/s12913-021-07076-x)
Supplement: Supplementary file 1 — Additional file 1. [file 12913_2021_7076_MOESM1_ESM.docx]

**Appendix 1: Examples of open-ended questions related to COVID-19 in phone interviews**

**Phone interviews with home care clients**

1. How has the COVID-19 pandemic changed home care services you receive? Would you describe how things were before and then after the pandemic?
2. How has COVID-19 changed your own home or day-to-day habits?
3. How has COVID-19 made you think about your and your family’s safety?
4. What precautions do you take now that you may not have done before the pandemic? What do you think are the most important precautions?
5. How has COVID-19 made you think about the safety of your home care aide? What precautions do you take now for your aide’s safety that you may not have done before the pandemic?
6. What are your top concerns related to COVID-19 and receiving home care?
7. Have you used telehealth/telemedicine during the pandemic? If yes, please tell us what you think about it? What forms of telehealth work for you the best?

**Phone interviews with home care aides**

1. How has the COVID-19 pandemic changed the day-to-day work you do? Would you describe how things were before and now during the ongoing pandemic?
2. What are your top concerns related to the pandemic now?
3. Do you care for positive or symptomatic COVID-19 clients?
4. How has COVID-19 changed your home care agency’s priorities? What are some of the safety changes, for you or for the clients that your agency has had to make during the pandemic?
5. Has your agency taught you about protecting yourself against getting sick with COVID-19? Please describe.
6. What is your agency’s policy on personal protective equipment (PPE)? Is your agency able to provide you with enough gloves, masks, and other supplies you need for COVID-19?
7. How has COVID-19 changed day-to-day life among your clients? What precautions do they take now that they may not have done before the pandemic?
8. Do your clients have the cleaning and disinfecting products that you need to clean their homes? Has the amount of time you need for cleaning changed since the pandemic began? Please explain.
9. Have you used telehealth technology during the pandemic with your clients? If yes, please tell us what you think about it?

**Phone interviews with home care agency directors and managers**

1. How has the COVID-19 pandemic changed your day-to-day work and priorities?
2. What are the top pandemic-related challenges your agency is dealing with?
3. Do you have adequate support for infection prevention training and access to PPE supplies for your staff?
4. What are some of the safety changes, for the aides or for the clients that your agency has had to make during the pandemic?
5. Does your agency think these changes are likely to continue when the pandemic has ended? Or has the pandemic changed some practices for the longer-term?
6. Will your home care practice be able to return to the way it was before the pandemic?
7. Is your agency caring for symptomatic COVID-19 clients? If not yet, is your agency prepared to care of clients, who are COVID-19 positive or persons under investigation?
8. How are clients and families responding to the COVID-19 crisis with regards to your aides/staff?
9. Are clients/families participating in the recommended infection prevention practices? If so, can you explain?
