# Supplementary material for: “It changed everything”: The safe Home care qualitative study of the COVID-19 pandemic’s impact on home care aides, clients, and managers
Source: BMC Health Serv Res. 2021 Oct 5;21:1055. doi: 10.1186/s12913-021-07076-x (PMC8491760; doi:10.1186/s12913-021-07076-x)
Supplement: Supplementary file 2 — Additional file 2. [file 12913_2021_7076_MOESM2_ESM.docx]

**APPENDIX 2 – Voluntary reporting of demographics for HC aides and clients**

Voluntary reporting of demographics – sex/gender, age, race, ethnicity – for HC aides and clients. Respondents could choose to answer all, some, or none of the following questions. Paper surveys were mailed to participants and included postage-paid return envelopes. No personal identifying information was collected, and responses remained anonymous. HC managers were asked similar questions in an online platform.

From Q1 and Q2, respondents could choose from “Female”, “Male”, or “Other”.

Q1. Biological sex

Q2. Gender identity

In Q3 and Q6, respondents were asked to provide a numerical response.

Q3. Age: I am _____ years old.

Q6-Aides. How long have you been working in home care?

Q6-Clients. How long have you been a client in home care?

Respondents were asked to select one response in Q4 to indicate “Hispanic or Latino” or “Not Hispanic or Latino”.

Q4. Ethnicity – mark one.

In Q5, respondents were asked to select all that apply from the following: “White”, “Black or African American”, “Asian”, “American Indian or Alaska Native”, “Native Hawaiian or Other Pacific Islander”, “Other race (please explain)”, with the option to enter free text if required.

Q5. Race – mark all that apply.

Q7 was an open-ended question in which respondents could provide one or more HC job titles (Q7-Aides) or HC services (Q7-Clients).

Q7-Aides. What is your current title for the home care job you do? (Example: home health aide, home care aide, homemaker)

Q7-Clients. What home care services do you receive? (Example: home health aide, home care aide, homemaker, other)
